# The Health Information Seeking Behavior of Punjabi Elders During the COVID-19 Pandemic in Canada

**DOI:** 10.1177/21501319241277576

**Published:** 2024-09-15

**Authors:** Amrit Thandi, Mohammad Z. I. Chowdhury, Nashit Chowdhury, Tanvir C. Turin

**Affiliations:** 1 University of Calgary, Calgary, AB, Canada

**Keywords:** Health Information Seeking Behavior, elderly populations, COVID-19, health communication, immigrants

## Abstract

Health Information Seeking Behavior (HISB) refers to the behavior and strategies used to attain, clarify, or confirm health information. The uptake of health information depends on system-level and individual-level factors. The purpose of the present study is to understand the sources from which Punjabi elders obtain COVID-19 vaccine-related information and their information seeking behavior. A cross-sectional survey was conducted among 391 Punjabi elders aged 50+ years in the Greater Toronto Area (GTA), Ontario. The survey questions included the need for COVID-19 vaccine information, the type of information sought, sources of information, and barriers to seeking information. Descriptive analysis was conducted using frequencies and percentages, and logistic regression was performed to understand the associations between participants’ sociodemographic characteristics and HISB. The results suggested that Punjabi elders are more likely to use informal sources and less likely to seek information from health professionals and government health and wellness websites. The results also suggested that most participants do not cross-check their information with other sources and are more likely to cross-check the information with family/friends, compared to credible care providers, across all demographics. Ultimately, there may be a need for stakeholders to collaborate to regulate the accuracy and type of health-information that is disseminated through media, and to tailor health communication to the health information seeking behavior of this population.

## Introduction

Health information may be used by the public to inform their decisions about lifestyle choices and healthcare experiences.^1^ Such information may be disseminated by a variety of sources, including government and non-government sources.^2^ By providing a wide array of health information, these types of health information sources in many countries have aimed to increase general and COVID-19-related health literacy.^3^ The World Health Organization (WHO) defines health literacy as the social and cognitive skills that enable individuals to access, comprehend, and utilize information in a health-promoting manner.^4^ Health literacy encompasses the ability to identify relevant, appropriate, and accurate information sources and then analyze, understand, and use the information for informed health decision-making.^5^

A major component of health literacy and health communication is Health Information Seeking Behavior (HISB), which refers to the behavior and strategies used to attain, clarify, or confirm health information.^6^ HISB considers the extent of the need for health information, the sources of information used and why these sources were used, and how the information is used.^6^ In the present literature, HISB has encompassed a variety of information sources that have varying degrees of reliability, including interpersonal and media sources. Further, the internet is now a predominant avenue through which many people find information,^1,7-9^ although studies have indicated that information-seekers often use a variety of sources to confirm their information. As such, understanding HISB of a population may lead to increased understanding of their health-related lifestyles, disease control, medical decision-making, and treatment.^10^

Despite the dissemination of health information, the uptake of this information depends on individual-level abilities and motivation to find it. Relatedly, there are several demographic determinants of HISB. Being of high income or having a high level of education are associated with positive health information seeking behaviors.^11-14^ Groups with these characteristics are also less likely to experience language barriers and may have better access to material resources.^1^ Further, the literature suggests that actively seeking or verifying health information decreases with age, meaning that the elderly are particularly vulnerable to low health literacy.^6,11,14^ This disparity between the ages is partly attributed to difficulties for the elderly in using telecommunications devices.^15^ Overall, there is a positive correlation between high income, high education level, health literacy, and good practice of HISB,^10,13,16^ meaning that socioeconomically disadvantaged groups do not have equitable levels of HISB, and lack the time and resources to seek health information.^1^

Consequently, certain populations including immigrants may be at risk for low health literacy due to cultural and linguistic barriers to good practice of HISB.^17,18^ Immigrants constitute 21.9% of the Canadian population and over 1.2 million immigrants settled in Canada from 2012 to 2016, accounting for 3.5% of the total population.^19^ According to the census in 2021, there are almost a million of Punjabi-speaking people live in Canada and an increasing number of South Asian immigrants in Canada. Some members of these populations have poor-self reported health compared to white populations, lower socioeconomic status, experience discrimination, transportation barriers, and also a lack of support from families and communities, in addition to limited English language proficiency, unfamiliarity with local services, and culturally insensitive care and information.^20^ Particularly, during the COVID-19 pandemic, prevailing misinformation and disinformation circulating through media, social networking sites (SNS), and word-of-mouth may have further exacerbated the challenges experienced by South Asian immigrant populations by contributing to low health literacy.^21^ It may have been difficult to determine the validity, reliability, or quality of the vast amount of available vaccine-related information.^22^ Elderly Punjabi immigrant populations in particular may have experienced worsened health literacy because of numerous barriers such as language, technological, and socioeconomic barriers while accessing COVID-19 and health-related information.^23,24^ Such barriers contribute to health disparities and health-literacy disparities for this population.^23,24^ To enable health-promoting decision-making among vulnerable elderly Punjabi immigrant populations, it is important to understand their HISB.

Against this backdrop, the present study aims to understand the sources from which elderly Punjabi immigrant populations obtain COVID-19 vaccine-related information, and their behaviors once the information is received. Given the challenges this group may experience when seeking health information, it is important to understand their HISB to provide them with additional support as needed and targeted information campaigns in the future. In the long term, identified sources of information can be used to educate and share accurate medical information to older Punjabi immigrant populations, ultimately lessening health disparities and improving health literacy in other contexts.

## Materials and Methods

### Theoretical Underpinning

This study is informed by Health Belief Model (HBM), Social Cognitive Theory (SCT), and HISB in the context of cultural competence and relevance.^6,25-27^ HBM provides the perspectives of the Punjabi elders in terms of their perceived severity of COVID-19 and the perceived benefits and barriers associated with vaccination influence and information sources through which they seek health information.^26^ SCT complements this by emphasizing the role of observational learning and personal, environmental, and social influence.^27^ This highlights how family members, community leaders, and peers within the Punjabi community in Canada may shape individual health information practices. The cultural competency and relevance of the public health communication shapes the HISB of the target population.^6^ The consideration of this aspects underscores the importance of cultural beliefs, language preferences, and the degree of acculturation in determining the accessibility and trustworthiness of health information sources.^25^ This explains the reliance of many immigrants on ethnic media and interpersonal communication over mainstream health sources, reflecting a preference for culturally resonant and linguistically accessible information.^28^ Together, these theoretical bases helped us design the survey instrument to capture the relevant information to have a comprehensive understanding of the information sources of the Punjabi elders and their behaviors upon receiving it, revealing a complex interplay between individual beliefs, social networks, and cultural context.

### Recruitment

Telephone and paper surveys were conducted among 391 Punjabi elders aged 50+ from elderly-serving community organizations in the Greater Toronto Area (GTA), Ontario. The survey language was English. The study was cross-sectional in design, and the survey questionnaire was developed based on theoretical understanding of HBM, SCT, and HISB in the context of cultural competence and relevance involving immigrant people. The study included elderly Punjabis who could communicate in English. At the time of the survey, the majority of information about COVID-19 was new and predominantly available in English. To have a comprehensive understanding of the information sources and HISB of Punjabi elders without being limited by language barriers, we determined that an English survey would be most effective. Additionally, we included a Punjabi-speaking team member who is connected with various elderly-serving community groups to conduct the surveys. This approach was intended to minimize any cultural limitations beyond language. Paper surveys were delivered and collected in person as needed. Participants were recruited using convenience sampling including at community events by community organizations with consent. Participants were mentally and physically sound to provide consent prior to participating in the survey.

Participants were asked demographic questions including their gender, age, marital status, education level, employment status, languages spoken, and length of stay in Canada since immigration. Afterward, participants were asked, their preferred sources of health information, their behaviors regarding the evaluation of a source. Participants’ preferred sources of health information were categorized as mainstream, social networking site (SNS) and interpersonal communication, specialized sources, and ethnic media. Mainstream media included TV, Newspaper, and the Radio. SNS and interpersonal communication included social networking sites such as Facebook, WhatsApp, Twitter, and non-specified SNS, and communication with friends/family, etc. Specialized sources included authorized medical/health websites, health professionals, governing health bodies, scientific journals, print media, and service-providing organizations. Ethnic media included the media in local languages which could take different forms such as TV, Radio, Newspaper, online news portals and others.

The inclusion criteria included being first-generation Punjabi person (people who were born outside Canada, had both parents born outside Canada), people who were able to effectively communicate in English; adults (>50 years old). The exclusion criteria included second-generation immigrants and individuals who had lived outside Canada during the COVID-19 period (April 2020-March 2021), as these individuals were likely to have missed out on Canadian vaccine and public health communications.

### Statistical Analyses

A descriptive analysis of the baseline variables was performed and expressed as numbers and percentages. Furthermore, a series of univariable and multivariable logistic regression analyses were performed to identify unadjusted and adjusted associations between different outcome variables and participant characteristics/exposure variables. The exposure variables included gender, age, level of education, length of stay in Canada, and self-rated health literacy. Outcome variables included information sources chosen, and source-checking behavior. Analyses were conducted using STATA version 15.1, and values of *P* < .05 were considered statistically significant.

## Results

### Demographic Characteristics

A total of 307 Punjabi elders (response rate 78.52%) over the age of 50 in the Greater Toronto Area completed the questionnaire. Participant characteristics are displayed in Table 1. Notably, most participants were male (66.12%), did not complete secondary/high school (62.54%), were retired (91.53%), and had been in Canada for at least 10 years (68.08%). The mean age was 72.09 years (SD = 7.18). Further, 90.50% of participants rated their overall health literacy as either very good (34.43%) or good (56.07%), and 97.72% of participants had a family doctor.

**Table 1.** Demographic Variables.

Demographic variables	Frequency	Percentage
*Gender*		
Male	203	66.12
Female	104	33.88
*Age*		
Male (mean, SD)	72.09 (7.18)	
Female (mean, SD)	68.49 ( 8.56)	
<65	56	18.24
≥65	251	81.76
*Relationship status*		
Married/Common law	305	99.35
Separated/Divorced	1	0.33
Widowed	1	0.33
Single	0	0
*Level of education*		
Completed Bachelor’s degree (4)	53	17.26
Completed some university/college courses (3)	2	0.65
Completed graduate/professional degree (5)	17	5.54
Completed secondary school or high school (2)	43	14.01
Did not complete secondary school or high school (1)	192	62.54
*Employment status*		
Full-time (5)	22	7.17
Part-time (4)	2	0.65
Unemployed and looking for work (3)	0	0
Looking after my home/family (2)	2	0.65
Retired from paid work (1)	281	91.53
*Languages spoken*		
English	1	0.33
Punjabi	306	99.67
*Length of stay in Canada since immigration in years*		
<10	98	31.92
≥10	209	68.08
*Do you have a family doctor?*		
Yes	300	97.72
No	7	2.28
*Overall health literacy rating (self-perception)*		
Excellent (5)	0	0
Very good (4)	105	34.43
Good (3)	171	56.07
Fair (2)	27	8.85
Poor (1)	2	0.66

### Sources of Information

The most frequently reported sources of COVID-19 vaccine-related information were grouped into mainstream media, SNS and interpersonal communication, specialized sources, and ethnic media (Table 2). Among the mainstream media, TV (39.74%), Radio (14.33%), and Newspaper (12.70%) were the most common. Within SNS and interpersonal communication, interpersonal communication (32.90%) was the primary source, followed by YouTube (3.91%), non-specified SNS (4.23%), Facebook (2.61%), WhatsApp (1.30%), and Twitter (0.33%). For specialized sources, health professionals (9.45%) and general internet webpages (8.79%) were notable, while government medical/health websites (1.30%), scientific journals (0.33%), governing health bodies (0.65%), and print media (0.33%) were less frequently used. Ethnic media was used by 8.79% of the participants.

**Table 2.** Information Sources of the Participants.

Information source (n = 307)	Frequency	Percentage
Mainstream media		
TV	122	39.74
Newspaper	39	12.70
Radio	44	14.33
SNS and interpersonal communication		
Interpersonal communication	101	32.90
YouTube	12	3.91
Facebook	8	2.61
WhatsApp	4	1.30
Twitter	1	0.33
Non-specified SNS	13	4.23
Specialized sources		
General Internet Webpages	27	8.79
Health professionals	29	9.45
Government medical/health websites	4	1.30
Governing health bodies	2	0.65
Scientific journals	1	0.33
Print media	1	0.33
Spiritual organizations	0	0.00
Community organizations	0	0.00
Service-providing organizations	0	0.00
Ethnic media	27	8.79

The logistic regression analysis (Table 3) suggests that females were less likely to use mainstream media (Unadjusted and Adjusted OR = 0.33; *P* < .001), SNS and Interpersonal Communication (Unadjusted OR = 0.39, *P* < .001; Adjusted OR = 0.41, *P* = .004) relative to males. All participants who had completed a bachelor’s degree were more likely to use mainstream media (Unadjusted OR = 2.62, *P* = .003; Adjusted OR = 2.52, *P* = .011), SNS and Interpersonal communication (Unadjusted OR = 2.46, *P* = .004; Adjusted OR = 2.30, *P* = .019) relative to participants who did not complete secondary or high school. Participants who had completed a graduate or professional degree had greater likelihood of using mainstream media (Unadjusted OR = 3.51, *P* = .023) and specialized sources (OR = 3.79, *P* = .023) compared to participants who did not complete secondary or high school; however, this was insignificant with the adjusted odds ratio. Similarly, participants who had stayed in Canada for 11 to 15 years were more likely to use mainstream media as a source of COVID-19 vaccine-related information (Unadjusted OR = 2.23, *P* = .04); however, this effect was insignificant once the adjusted odds ratio was calculated. We did not observe any statistically significant associations for the other variables.

**Table 3.** Multivariable Logistic Regression Model for Preferred Information Sources.

	Mainstream media				Specialized Sources				SNS and Interpersonal Communication				Ethnic Media			
Different Types of Information Sources	Unadjusted OR [95% CI]	*P*-value	Adjusted OR [95% CI]	*P*-value	Unadjusted OR [95% CI]	*P*-value	Adjusted OR [95% CI]	*P*-value	Unadjusted OR [95% CI]	*P*-value	Adjusted OR [95% CI]	*P*-value	Unadjusted OR [95% CI]	*P*-value	Adjusted OR [95% CI]	*P*-value
*Gender*																
Male	Reference		Reference		Reference		Reference		Reference		Reference		Reference		Reference	
Female	0.33 [0.20-0.55]	<.001	0.33 [0.18-0.58]	<.001	0.45 [0.19-1.07]	.071	0.50 [0.18-1.36]	.173	0.39 [0.23-0.66]	<.001	0.41 [0.22-0.75]	.004	0.53 [0 0.21 - 1.36]	.186	0.69 [0.25-1.91]	.472
*Age*																
50-55	Reference		Reference		Reference		Reference		Reference		Reference		Reference		Reference	
56-60	0.48 [0.13-1.80]]	.275	0.49 [0.11-2.16]	.348	0.39 [0.03-4.78]	.465	0.28 [0.02-4.44]	.365	0.38 [0.09-1.51]	.167	0.36 [0.08-1.67]	.193	—		—	
61-65	0.67 [0.21-2.14]	.495	0.52 [0.14-1.92]	.324	0.97 [0.16-5.89]	.972	0.80 [0.11-5.94]	.825	0.75 [0.23-2.41]	.629	0.57 [0.16-2.11]	.402	0.32 [0.03-3.79]	.369	0.39 [0.03-4.82]	.46
66-70	1.02 [0.35-2.92]	.977	0.77 [0.23-2.66]	.684	1.88 [0.39-9.10]	.436	1.67 [0.27-10.32]	.582	0.71 [0.25-2.05]	.525	0.59 [0.17-2.01]	.4	1.5 [0.30-7.48]	.621	1.65 [0.31-8.75]	.558
71-75	0.99 [0.35-2.81]	.992	0.84 [0.25-2.88]	.782	0.45 [0.08-2.52]	.361	0.41 [0.06-3.03]	.382	0.84 [0.30-2.37]	.74	0.81 [0.24-2.73]	.729	1.88 [0.39-8.98]	.428	2.22 [0.45-11.09]	.33
76-80	0.52 [0.16-1.65]	.266	0.38 [0.10-1.44]	.154	0.42 [0.05-3.24]	.403	0.32 [0.03-3.07]	.326	0.35 [0.10-1.17]	.089	0.27 [0.07-1.04]	.058	0.61 [0.08-4.66]	.635	0.61 [0.08-4.77]	.637
81-85	0.53 [0.15-1.88]	.329	0.48 [0.11-2.03]	.319	1.97 [0.33-11.63]	.453	1.91 [0.25-14.41]	.529	0.80 [0.23-2.81]	.732	0.80 [0.19-3.29]	.76	—		—	
85+	0.71 [0.14-3.61]	.681	0.57 [0.09-3.48]	.539	0.94 [0.07-11.99]	.96	0.77 [0.05-12.43]	.856	0.56 [0.10-3.02]	.502	0.54 [0.08-3.44]	.513	—		—	
*Level of education*																
Did not complete secondary school or high school	Reference		Reference		Reference		Reference		Reference		Reference		Reference		Reference	
Bachelor’s degree	2.62 [1.39-4.92]	.003	2.52 [1.24-5.10]	0.011	1.16 [0.44-3.07]	.762	1.00 [0.34-2.97]	.997	2.46 [1.33-4.58]	0.004	2.30 [1.15-4.61]	.019	0.79 [0.26-2.44]	.681	1.00 [0.30-3.34]	1
Some university or college	1.46 [0.09-23.72]	.79	0.78 [0.04-15.52]	0.869	—		—		—		—		—		—	
Graduate or professional degree	3.51 [1.19-10.35]	.023	2.48 [0.79-7.78]	0.12	3.79 [1.21-11.93]	.023	2.95 [0.75-11.56]	.12	2.48 [0.91-6.73]	0.076	1.86 [0.63-5.50]	.259	1.29 [0.27-6.09]	.749	1.99 [0.36-11.05]	.43
Completed secondary school or high school	1.27 [0.65-2.47]	.48	1.18 [0.57-2.42]	0.66	1.20 [0.42-3.41]	.735	1.28 [0.39-4.18]	.685	1.74 [0.89-3.42]	0.107	1.67 [0.80-3.49]	.171	0.73 [0.20-2.58]	.62	1.02 [0.27-3.92]	.975
*Length of stay in Canada*																
0-5	Reference		Reference		Reference		Reference		Reference		Reference		Reference		Reference	
6-10	1.02 [0.48-2.19]	.954	1.12 [0.49-2.59]	0.786	—		—		0.64 [0.28-1.44]	0.277	.69 [.29-1.67]	.414	0.93 [0.13-6.82]	.94	0.63 [0.08-4.87]	.659
11-15	2.23 [1.04-4.79]	.04	2.22 [0.95-5.19]	0.066	2.21 [0.77-6.34]	.138	2.15 [0.68-6.82]	.193	1.86 [0.87-3.99]	0.111	2.11 [.91-4.88]	.083	4 0.00 [0.81-19.78]	.089	2.95 [0.57-15.37]	.199
16-20	1.10 [0.58-2.09]	.768	1.03 [0.51-2.11]	0.926	0.98 [0.36-2.66]	.97	0.89 [0.30-2.67]	.834	1.01 [0.52-1.96]	0.965	.97 [.47-2.00]	.935	2.98 [0.66-13.49]	.157	2.40 [0.49-11.65]	.277
*Level of health literacy*																
Low	Reference		Reference		Reference		Reference		Reference		Reference		Reference		Reference	
High	1.30 [0.60-2.82]	.509	0.96 [0.40-2.30]	0.928	0.59 [0.21-1.65]	.311	0.52 [0.16-1.70]	.281	0.86 [0.39-1.86]	0.697	0.69 [0.29-1.66]	.406	0.57 [0.18-1.77]	.33	.58 [.17-2.03]	.396

### Information Seeking Behavior

**Figure 1.**
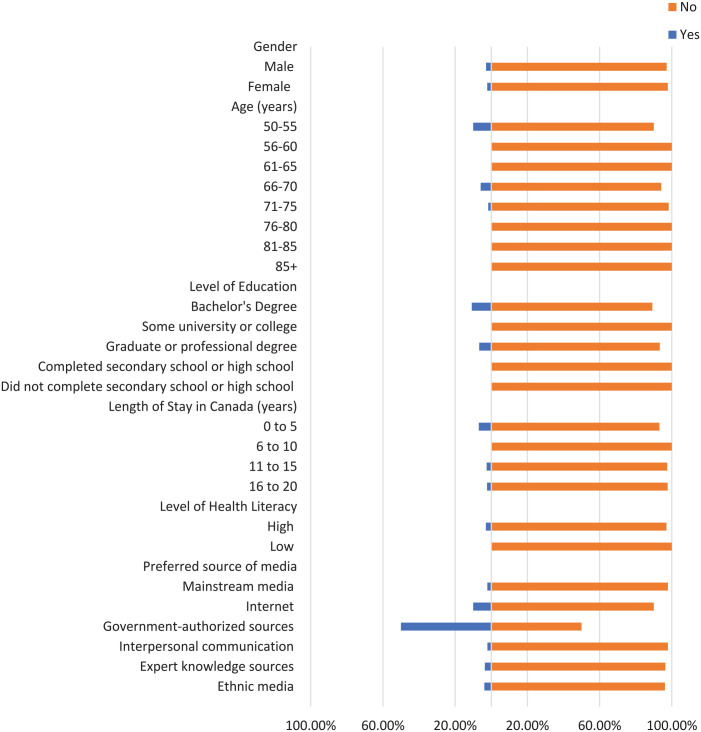
Characteristics of the participants who checked domain name.

**Figure 2.**
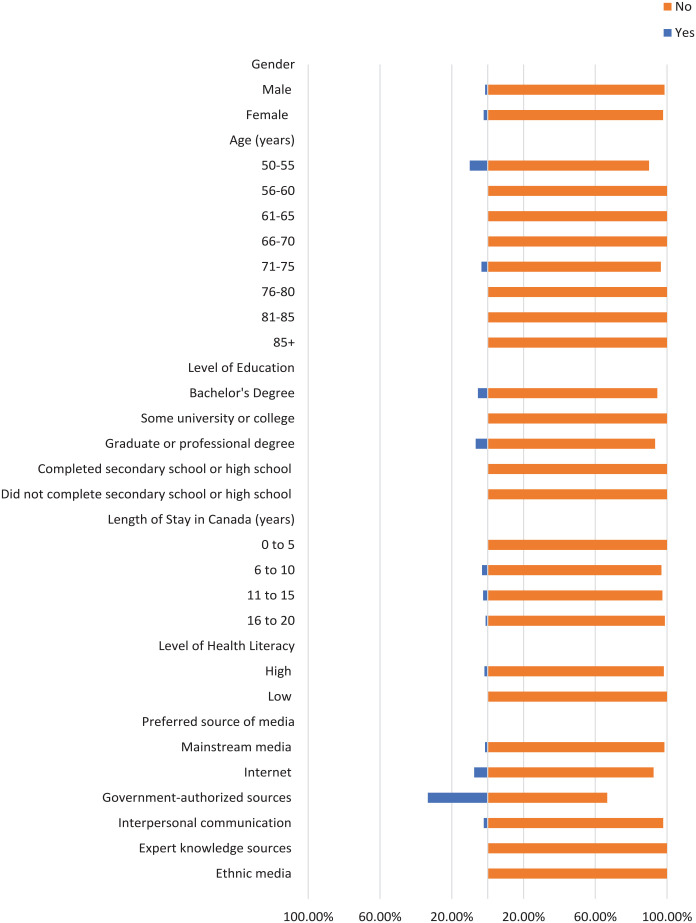
Characteristics of the participants who checked original source.

**Figure 3.**
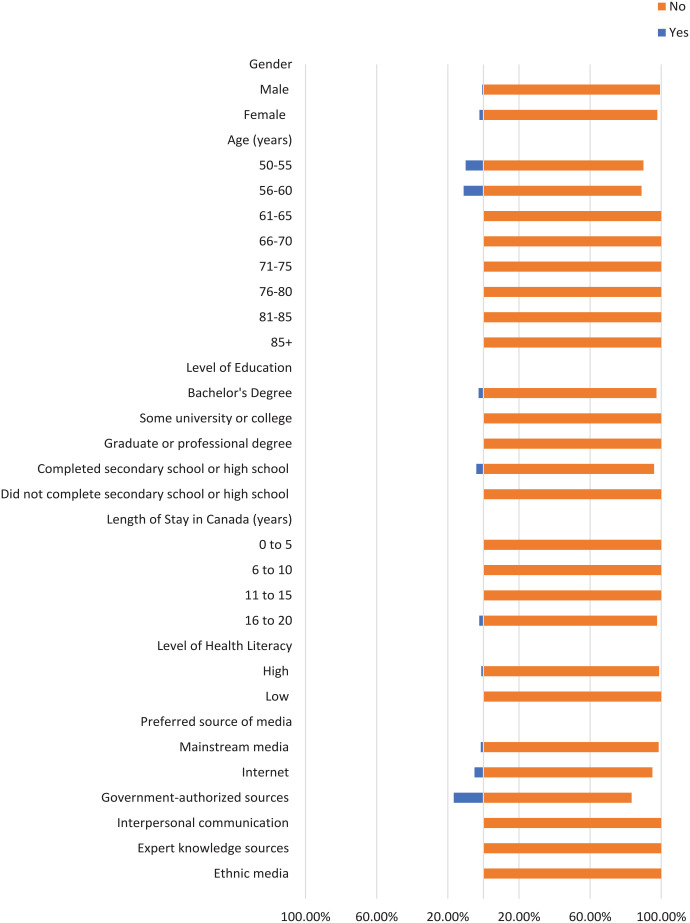
Characteristics of the participants who checked credentials of source.

**Figure 4.**
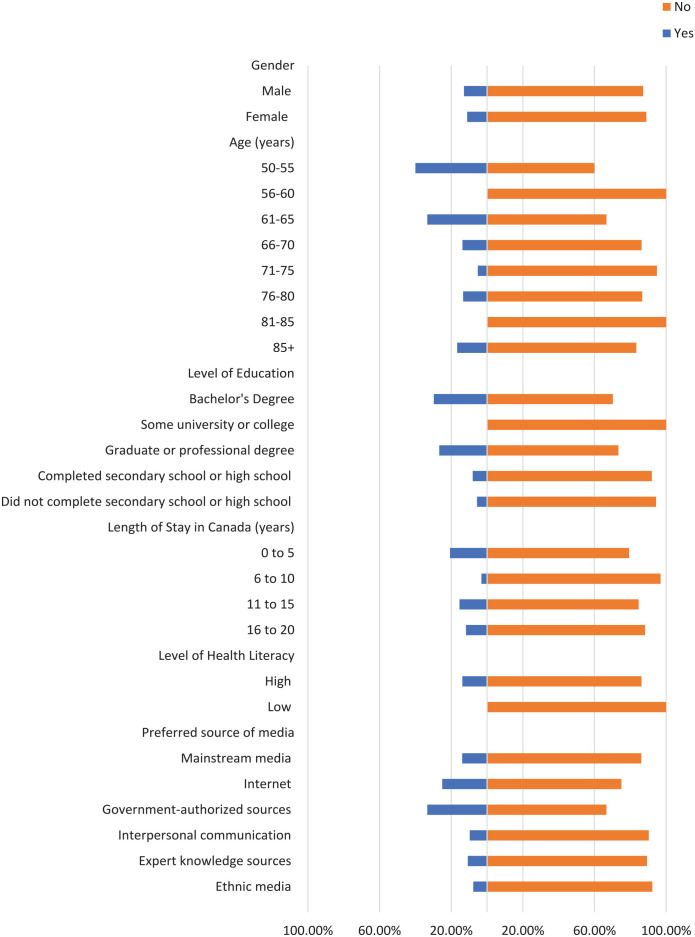
Characteristics of the participants who cross-checked information with other sources.

**Figure 5.**
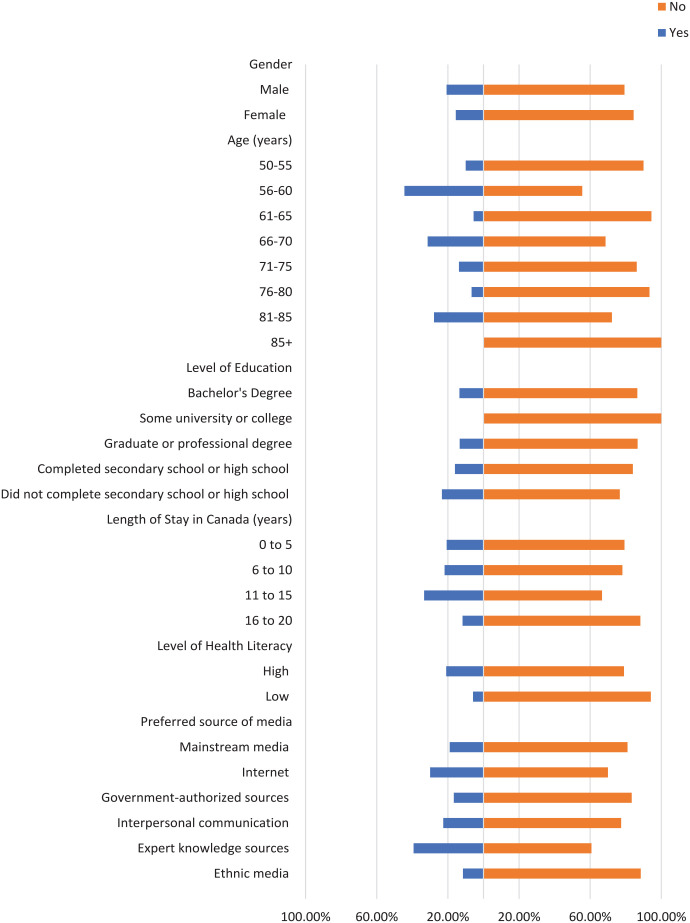
Characteristics of the participants who cross-checked information with care providers.

**Figure 6.**
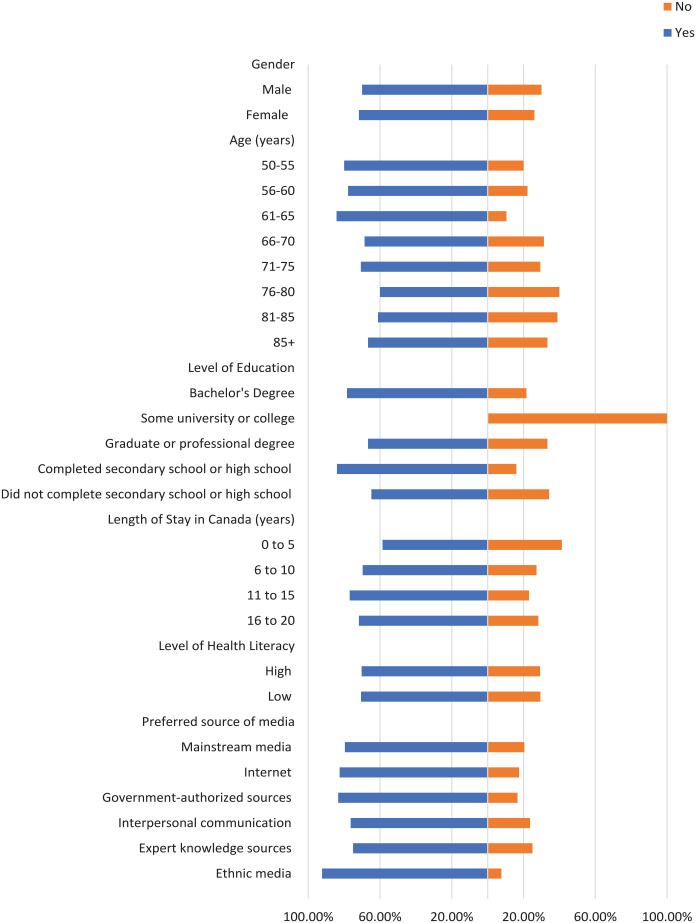
Characteristics of the participants who cross-checked information with friends/family.

Most participants did not check the domain name of their sources where applicable, the original source of information, or the credentials of the source (Figures 1-3). Moreover, most participants did not cross-check their information with other similar sources (Figures 4 and 5), regardless of demographics (male: 87.14%, female: 88.89%), or care providers (male: 79.29%, female: 84.44%). However, more individuals cross-checked their information with friends/family (male: 70.00%, female: 71.74%; Figure 6).

**Figure 7.**
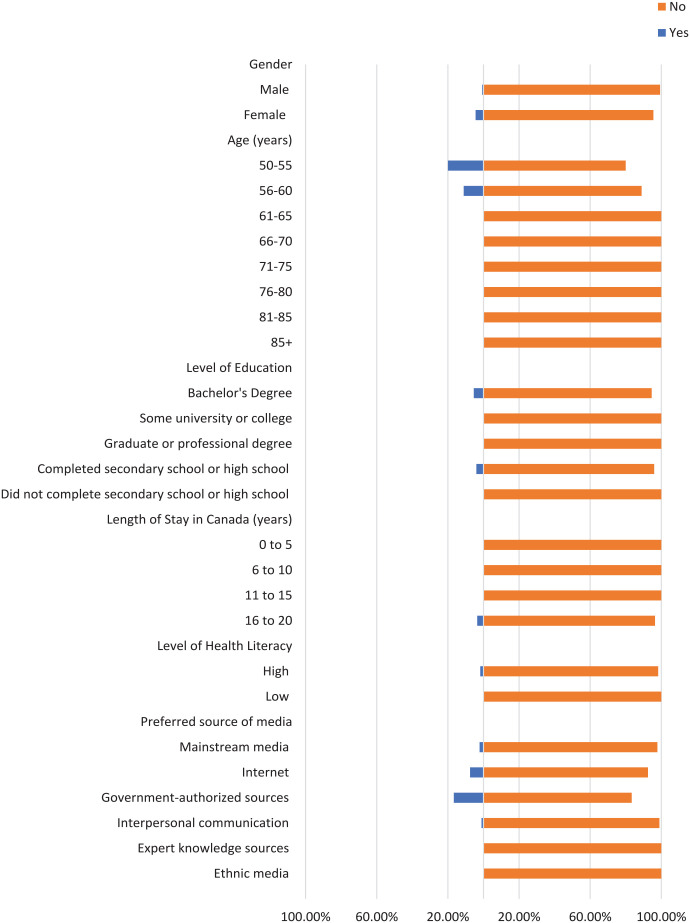
Characteristics of the participants who checked if information matches religious/cultural beliefs.

**Figure 8.**
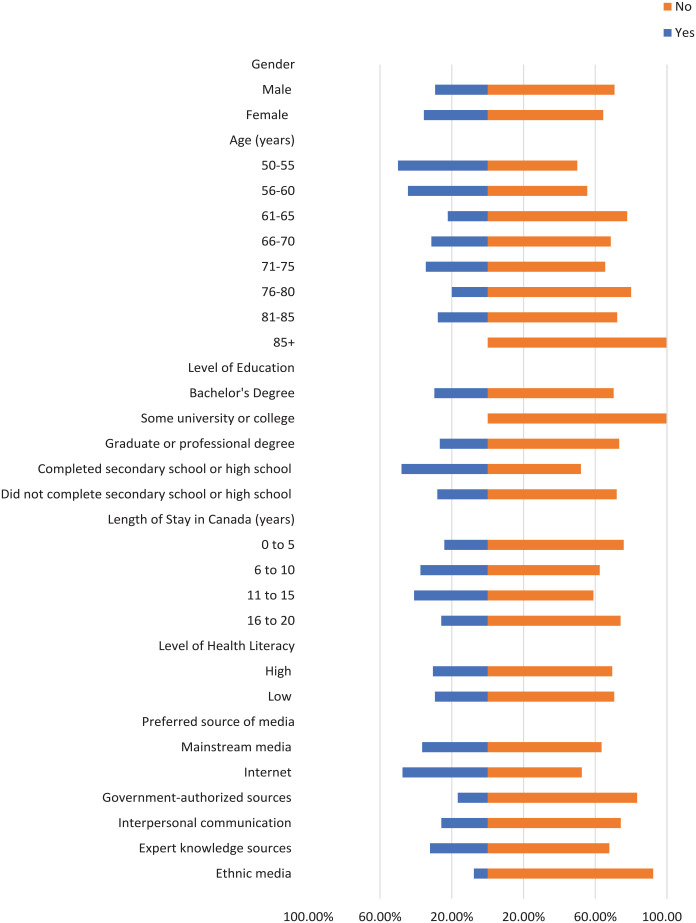
Characteristics of the participants who believed when person shares from own experience.

When it comes to checking and cross-checking information, the percentages were similar between males and females. However, the percentage of females (4.44%) who checked if the information matched their religious/cultural beliefs (Figure 7) was higher compared to males (0.71%). A similar pattern was observed in terms of believing information when a person shares it from their own experience (male: 29.29%, female: 35.56%; Figure 8).

The percentage of individuals aged 50 to 55 years who cross-checked information with other sources (40.00%) is greater than other age groups, and the percentage of individuals aged 56 to 60 who cross-checked information with their care providers (44.44%) is also greater (Figures 4 and 5). Participants with high school as their highest education were more likely to cross-cross information with family/friends (84.00%) and believe information when it is shared from a person’s own experience (48.00%), compared to participants with a higher level of education (Figures 6 and 8). Notably, participants with a bachelor’s degree had the highest percentage of checking the domain name (10.81%), the original source (5.41%), and credentials of the source (2.70%) compared to other education levels (Figures 1-3).

Participants with a low level of health literacy had a lower percentage of cross-checking information with other sources (0.00%) or care providers (5.88%) compared to those with a high level of health literacy (13.77% and 20.96%, respectively; Figures 4 and 5). The percentage of individuals who used government-authorized sources and checked the original source of their information (33.33%) was higher compared to those using other preferred sources of media (Figures 2 and 3).

## Discussion

The aim of the present study was to understand the sources from which elderly Punjabi immigrant populations obtain COVID-19 vaccine-related information, their reasons for choosing their preferred sources, the evaluation of these sources, and the barriers they experienced when accessing vaccine-related information, using a cross-sectional survey. The results of our study indicate significant differences in information sources, reasons for choosing sources, and barriers experienced, in relation to demographic characteristics.

The present study indicated that the most frequently used sources of COVID-19 vaccine-related information included Television and Interpersonal Communication, with participants being less likely to use health professionals and government medical/health websites. In a study about immigrant parents’ preferred information sources for health information about their child, the most preferred sources included the Internet, then interpersonal communication, and lastly healthcare professionals.^29^ The results of the present study support interpersonal communication being a relatively commonly preferred source of information among Punjabi elders, compared to healthcare professionals. Our results also support the importance of interpersonal communication in receiving COVID-19 related information for Punjabi elders, as demonstrated in a recent qualitative study about COVID-19 public health communication among South Asian communities in Canada.^30^ The disparity between immigrant parents’ increased preference for the internet compared to elderly South Asian immigrants may be due to limited knowledge about technology use for the elderly and a generational gap.^18^

Further, there were demographic differences in participants’ preferences for health information sources, which suggests that health information dissemination should consider the diversity of the population. Female participants were found to use mainstream media as their information source less commonly than their male counterparts. While use of internet sources such as SNS were less frequent among the participants, females were even less likely to use these sources. This finding aligns with previous literature suggesting that men traditionally have had higher interest in technology, however it contradicts more recent findings of women being more likely to use the internet for HISB.^31^ Further, the literature indicates that the elderly may experience barriers to using the internet as an information source such as individual abilities, social expectations, and technology barriers.^32^ Further, the elderly may perceive healthcare providers to be the most acceptable source of health information, in comparison to the internet. Among South Asian immigrants in Edmonton, other predictors of not owning a device to access the internet include: being low education, having immigrated to Canada within the past 5 years, and preferring information about health in languages other than English, in addition to having chronic disease.^33^ Given that the current study population exhibited these characteristics, they may be at increased likelihood of not owning a device and therefore not using the internet frequently as a source of information.^33^ Further, in the present study, participants who had completed a bachelor’s degree were more likely to use mainstream media and SNS relative to participants who did not complete secondary or high school. This result is supported by the literature which suggests that populations with higher education are more likely to use online and SNS sources for health information and also have higher health literacy.^34,35^ Overall, it is important to consider the unique challenges experienced by Punjabi elders, particularly vulnerable low socioeconomic groups who are at greater disadvantage of poor health, when disseminating public health information.

Moreover, the present study addressed a gap in the literature by exploring participants’ evaluation skills of the sources. The results suggested that most participants do not cross-check their information with other sources and more commonly cross-check with family/friends, compared to care providers, across all demographics. Previous studies demonstrates that cross-checking information is an important part of information seeking behavior online and can help make informed decisions.^35,36^ Thus, it is important to ensure the information being communicated between Punjabi elders is accurate. A study of South Asian communities’ interpersonal communication during the pandemic in Ontario suggested that although interpersonal communication is important, it may perpetuate the spread of misinformation through platforms such as WhatsApp.^30^ Coupled with this finding, the results of our study suggest that an effective means of spreading accurate information among the elderly Punjabi community may be to monitor the information shared on social media platforms. Further, our results indicated that most participants did not check the domain name of their sources if they used the internet, nor did they check the original source of information, or the credentials of the source. This may contribute to the spread of misinformation during times of emergency such as the pandemic, given that the accuracy or reliability of the source is not verified. This gap in evaluation by Punjabi elders suggests there may be a need for health source evaluation initiatives, or easier accessibility to information about sources on websites that are not government websites.

### Strengths and Limitations

The strengths of this study include its focus on Punjabi elders in the Greater Toronto Area (GTA). Current literature does not address HISB in this population subgroup, so this study adds value to the literature by addressing their unique preferred sources of health information, their HISB, and barriers to information. Another strength is the study’s comprehensive questionnaire that addressed all aspects of HISB including barriers to achieving good HISB. Consequently, the findings of the study can holistically address Punjabi elders’ HISB and provide insight into how larger structures in society like governments can better target information to this group. Given that the elderly Punjabi population is increasing in Canada and is at greater risk for poor health outcomes, it is important to address this gap. Limitations of this study include its sample size which may not provide adequate statistical power. Moreover, males were overrepresented in the study population compared to females and the study used a convenience sampling approach suggesting that the findings may not be generalizable to all Punjabi elders in Canada. Further, some first-generation immigrants may not have participated in the study because the survey was only available in English.

## Conclusion

Overall, the purpose of the present study was to gain insight into the Health Information Seeking Behavior of Punjabi elders in the GTA. The results of the study suggest that there may be a need to monitor the accuracy of information disseminated through media and use targeted health information dissemination strategies that address the need and health information seeking behavior by this population, to ultimately promote better health outcomes.
